# Plant Growth and Morphogenesis under Different Gravity Conditions: Relevance to Plant Life in Space

**DOI:** 10.3390/life4020205

**Published:** 2014-05-16

**Authors:** Takayuki Hoson

**Affiliations:** Department of Biology, Graduate School of Science, Osaka City University, Sumiyoshi-ku, Osaka 558-8585, Japan; E-Mail: hoson@sci.osaka-cu.ac.jp; Tel./Fax: +81-6-6605-2577

**Keywords:** automorphogenesis, cell wall, growth, microgravity, plants, space conditions

## Abstract

The growth and morphogenesis of plants are entirely dependent on the gravitational acceleration of earth. Under microgravity conditions in space, these processes are greatly modified. Recent space experiments, in combination with ground-based studies, have shown that elongation growth is stimulated and lateral expansion suppressed in various shoot organs and roots under microgravity conditions. Plant organs also show automorphogenesis in space, which consists of altered growth direction and spontaneous curvature in the dorsiventral (back and front) directions. Changes in cell wall properties are responsible for these modifications of growth and morphogenesis under microgravity conditions. Plants live in space with interesting new sizes and forms.

## 1. Introduction

A plant must be of the proper size and form to perform efficient physiological and biochemical processes. The regulation of growth for size and of morphogenesis for form, thus, is very important for plant life. Because the form of the whole plant reflects the sum of the rates and directions of growth for different parts, the growth and morphogenesis are tightly associated with each other.

Plant growth and morphogenesis are fundamentally regulated by a genetic program, as is the case in animals. However, plants are also surrounded by a great variety of environmental signals, such as light, gravity, temperature, and water, which strongly influence their processes of growth and morphogenesis. Gravity is unique among these environmental signals, in that it is always present in a constant direction and magnitude on earth. Plants have utilized gravity as the most stable and reliable signal for their survival over the course of evolution. Microgravity is one of the most significant features of orbital flight in space. Thus, we predict that plant growth and morphogenesis will be greatly modified under space conditions.

## 2. Growth and Morphogenesis on a Rotating Clinostat

**Figure 1:**
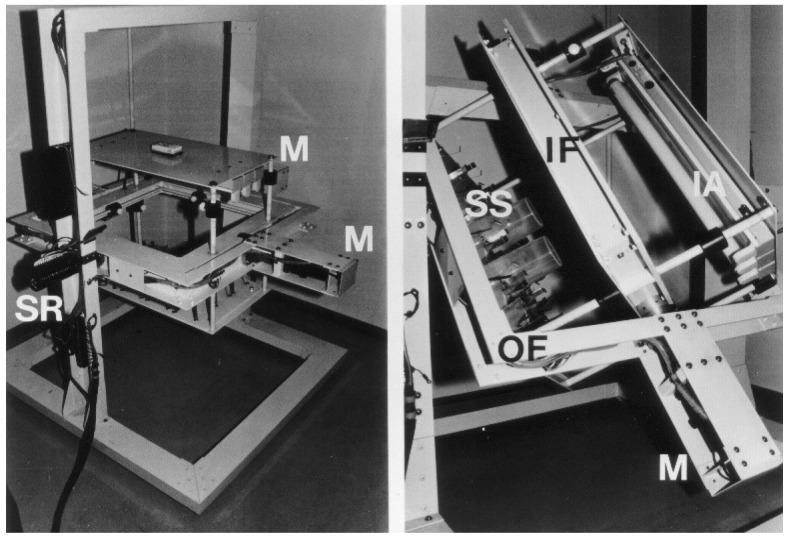
Three-dimensional (3-D) clinostat. Left, initial position. The sample stage (SS) is rotated about a horizontal axis in a conventional clinostat. Right, after 3-D rotation with two motors (M) placed at right angles. IA, illumination apparatus; IF, inner frame; OF, outer frame; SR, slip rings.

True microgravity conditions can be produced by free fall or parabolic flight on earth. However, the microgravity obtained by these methods is generally too brief to induce obvious changes in plant growth and morphogenesis. Therefore, a clinostat with a horizontal axis, which compensates for the unilateral influence of gravity, has been widely used to simulate microgravity (Figure 1). A horizontal clinostat is convenient, but it often causes undesired variations and side effects, likely due to the flow of air or solution in a constant direction and the chronic stimulation of the lateral sides of the materials [1,2]. To avoid such effects, we developed a three-dimensional (3-D) clinostat equipped with two rotation axes placed at right angles [3]. Plant materials were continuously rotated in three dimensions on this clinostat by changing the rates of rotation of the two motors at random, and the changes in growth were analyzed. The results have indicated that growth and growth-regulating parameters are not significantly influenced by clinostat rotation, at least for short durations, in most plant materials [1,3], although exaggerated growth due to modifications of cellular structure may occasionally be induced by long-term rotation [2,4,5]. These observations may result from the inability of the clinostat to eliminate static gravistimulation (magnitude of gravity) as the device counters only dynamic gravistimulation (direction of gravity) [4,5].

On the other hand, plant morphogenesis is greatly modified on a clinostat. Since the studies of Sachs and Pfeffer more than a century ago, spontaneous morphogenesis, termed automorphogenesis, has been observed in various plant materials on the clinostat [1,3,6]. Generally, automorphogenesis consists of altered growth direction and/or spontaneous (automorphic) curvature of organs. Spontaneous curvature occurs in growing regions in the direction explained by the presence of dorsiventrality (having distinct back and front structures) [3,5,7]. The angle of automorphic curvature increases with organ growth in the early growth phase on the clinostat, but seedlings lose dorsiventrality and show a radial form after approximately a week of growth. Automorphic curvature differs in various parameters from tropistic curvatures [1]. For instance, the rate of automorphic curvature is only one-tenth of that of gravitropic curvature.

Plant roots on the 3-D clinostat first grow along the direction of the tips of the root primordia and then in random directions [3]. This transition of growth pattern occurs in the early growth phase in some species, such as maize and pea, but in the late growth phase in other species, such as rice and garden cress. Clinorotated roots also show automorphic curvature. The automorphic curvature of roots occurs in random directions, without any dorsiventrality [8]. Thus, the automorphogenesis of roots on the 3-D Clinostat also consists of altered growth direction and spontaneous curvature.

The mechanisms by which automorphic curvature is induced on the 3-D clinostat have been studied with maize and rice seedlings. The rate of plant cell growth is most directly regulated by the osmotic potential of the cell sap and the mechanical extensibility of the cell wall. Previous studies have shown the direct cause of differential growth in clinorotated coleoptiles and roots to be the difference in cell wall extensibility between the convex and concave sides, not the difference in cellular osmotic potential [1,7]. A number of differences in the metabolic turnover of the cell wall constituents have been detected between the two sides [1,7]. Changes in microtubule orientation may also be involved in the automorphic curvature of shoot organs. The cortical microtubules of epidermal cells on the convex side are oriented more transversely than those on the concave side in clinorotated rice coleoptiles [9]. These changes preceded the automorphic curvature when rice seedlings were transferred from a stationary orientation to the 3-D clinostat [1].

**Figure 2:**
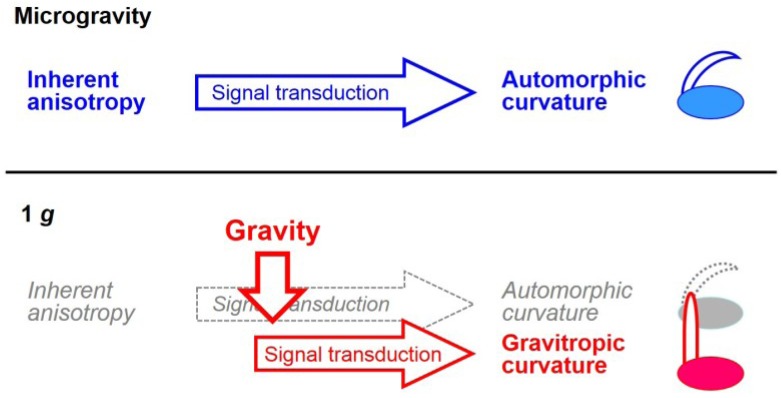
The mechanism inducing automorphic curvature. Under microgravity conditions, the inherent structural and physiological anisotropies of plant organs induce spontaneous automorphic curvature, whereas in the presence of gravity, the organs are forced to grow along the vector of gravity, leading to gravitropic curvature.

Clear structural anisotropy exists between the back and front sides of young shoot organs [1,7]. For instance, the back, convex side of a rice coleoptile consists of small, extensible cells, as compared with its front, concave side. In the presence of a gravity vector, this difference in growth capacity is likely diminished, and the shoot organs are forced to grow upward evenly along the vector; in the absence of this vector, the organs show automorphic curvature following their inherent anisotropy (Figure 2). The involvement of reduced polar auxin transport in automorphogenesis has been demonstrated in pea seedlings grown on a clinostat [10].

## 3. Growth and Morphogenesis under Submergence

Growth of plant seedlings is not significantly influenced by clinostat rotation, as mentioned above, and the magnitude of gravitational acceleration must be changed to examine the effects of gravity on growth processes. Water immersion has been used to simulate microgravity in the development of space science and technologies. Submergence is recognized as a kind of microgravity, because the apparent strength of gravity, as measured by the weight of materials, is reduced due to buoyancy, even if gaseous and other factors are also different and no changes in cellular events occur underwater [1,11].

**Figure 3:**
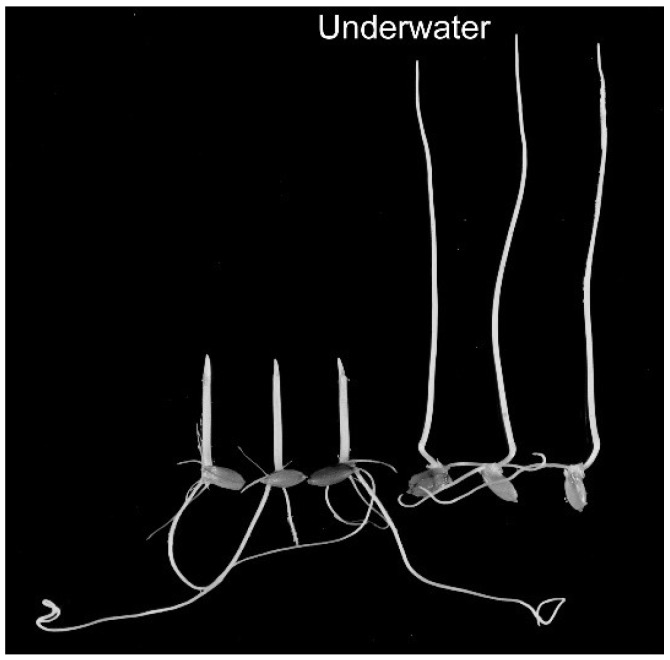
Rice seedlings grown in air (with only roots immersed in water) or underwater. Submergence induces the stimulation of elongation and the suppression of lateral expansion in rice coleoptiles.

Land plants are generally unable to survive underwater. However, aquatic or semi-aquatic plants such as rice can grow for long periods under conditions of submergence. Rice coleoptiles elongate rapidly and are growing slender when growing underwater (Figure 3), as submergence induces the stimulation of elongation and suppression of lateral expansion [1,12]. Because the modification of growth anisotropy is suppressed only partially by air bubbling, the microgravity effect due to buoyancy, in addition to gaseous factors, may be involved. Similar changes have been observed in deepwater rice and *Regnillidium* grown underwater [1,12].

The cell wall extensibility of rice coleoptiles grown under submergence is higher than that of coleoptiles grown in air [1,12]. Submergence also causes diverse changes in the levels and metabolism of the cell wall constituents of rice coleoptiles. These changes in cell wall properties may be responsible for the modifications of growth and morphogenesis observed in plant seedlings under submergence.

## 4. Growth and Morphogenesis under Hypergravity Conditions

**Figure 4:**
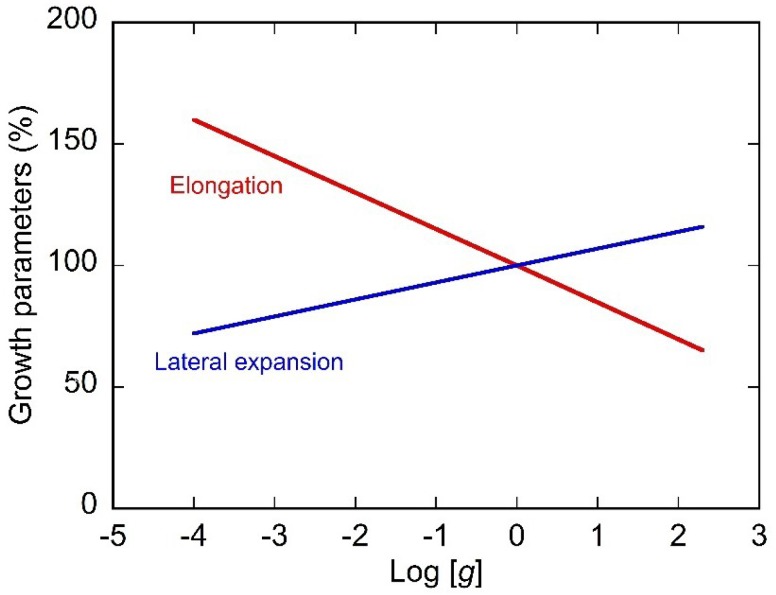
Dose-response relationship between the magnitude of gravity and growth parameters. The magnitude of gravity is plotted on a logarithm basis. Growth parameters vary in proportion to the logarithm of the magnitude of gravitational acceleration between 1 × 10^−4^ *g* (the average magnitude of gravity during Space Shuttle flight and on the International Space Station) and 300 *g*.

Another practical method to modify the magnitude of gravitational acceleration is centrifugal hypergravity. Waldron and Brett first analyzed the effects of hypergravity on the growth and cell wall compositions of pea seedlings [13]. The responses of various other plant materials to centrifugal acceleration have since been analyzed, and hypergravity has been shown to decrease the longitudinal growth rate of various shoot organs and roots [1,13,14]. Hypergravity not only suppresses elongation growth but promotes the lateral expansion of organs [15,16]. Plant organs are highly resistant to gravitational acceleration, and growth parameters vary in proportion to the logarithm of the magnitude of gravitational acceleration up to 300 *g* (Figure 4). Linear dose-response relationships to the logarithm of a dose are common among biotic responses in which proteinaceous signal receptors, such as photoreceptors and hormone receptors, are involved. When plant seedlings grown under hypergravity conditions at 300 *g* for several hours are transferred to 1 *g* conditions, the growth rates recover fully within a couple of hours, indicating that the effects of gravitational acceleration on growth properties are prompt and reversible [1].

Regarding the cell wall properties that regulate the growth rate, hypergravity has been shown to decrease the cell wall extensibility [1,17]. Hypergravity also induces the accumulation of cell wall constituents, polymerization of certain matrix polysaccharides due to suppression of breakdown, stimulation of cross-link formation, and increase in cell wall pH, depending on the magnitude of the gravitational acceleration; all of these factors decrease the cell wall extensibility. Thus, the modifications of the growth and cell wall properties of plant seedlings caused by hypergravity are opposite to those caused by submergence.

Cortical microtubules are likely involved in the modification of growth anisotropy by hypergravity through the regulation of the orientation of cellulose microfibrils. The expressions of most α- and β-tubulin genes are upregulated by hypergravity [18,19]. In the growing regions of stem organs at 1 *g*, cells with transverse cortical microtubules are predominant. As gravitational acceleration increases, the percentage of cells with transverse microtubules decreases, whereas that of cells with longitudinal microtubules increases [15]. On the other hand, the stem organs of tubulin mutants are shorter and thicker than those of the wild type and display helical growth at 1 *g*. The degree of this twisting phenotype is high under hypergravity conditions [20]. Thus, hypergravity intensifies the morphological defects of certain mutants related to gravity responses.

## 5. Growth and Morphogenesis under Microgravity Conditions in Space

### 5.1. Growth

The ground-based experiments used to clarify the effects of gravity on plant growth and morphogenesis all possess specific fundamental weaknesses, as mentioned above. Thus, studies must be conducted under true microgravity conditions in space. Previous opportunities for space experiments have been limited, but the situation was greatly improved by the initiation of scientific operations on the International Space Station in 2008.

From the results of experiments using water submergence and centrifugal hypergravity, the elongation of seedlings was hypothesized to be stimulated under true microgravity conditions in space. The growth rates of various plant organs have been measured in space, but these results have been mixed, likely due to variations and differences in temperature, light, water, gaseous parameters, and sample storage conditions [1]. For example, plant materials were often exposed to light during the experiments, which modified their growth so as to cancel the effects of microgravity; light causes similar changes in cell wall properties as gravity does [1,12] and acts as a substitute for gravity in growth regulation [21]. Additionally, chemicals that accumulated in the cabin of the spacecraft, such as ethylene, strongly influenced plant growth [22]. In the Space Shuttle STS-95 mission, we conducted an experiment, designated as RICE, in careful consideration of these conditions, to clarify the changes in the growth and cell wall properties, as well as morphogenesis, of rice and Arabidopsis seedlings during spaceflight. Importantly, all experimental procedures were conducted in the dark. The results confirmed the stimulation of elongation in space for both rice coleoptiles [23] and Arabidopsis hypocotyls [24]. Growth stimulation in Arabidopsis seedlings has also been observed in other space experiments [25]. Additionally, the elongation of rice roots is stimulated significantly in space, and the degree of stimulation increases with aging [26]. When the longitudinal growth of inflorescence stems was compared between microgravity and 1 *g* conditions, the stems were longer and their growth rate was higher under microgravity conditions than under both control conditions [27]. Because no clear differences were detected in the length or the growth rate of the stems between the ground and in-orbit 1 *g* controls, the stimulation of inflorescence growth may be caused by microgravity, not by spaceflight.

We have analyzed both the mechanical and chemical properties of the cell walls of space-grown seedlings [23,24]. The cell wall extensibility of rice coleoptiles and Arabidopsis hypocotyls decreased as the growth period increased and was always higher in space-grown seedlings than in the controls. Rice coleoptiles and Arabidopsis hypocotyls grown under microgravity conditions had lower levels of cellulose and matrix polysaccharides per unit length than did the controls, indicating that microgravity decreased the cell wall thickness. The space-grown materials also had lower molecular masses of the matrix polysaccharides, caused by the elevated activity of the cell wall enzymes involved in their breakdown. Thus, under microgravity in space, the metabolic turnover of the cell wall constituents of plant seedlings is stimulated, thereby increasing the cell wall extensibility and stimulating elongation [1,17]. Furthermore, the result that growth of the inflorescence stems of a tubulin mutant was restored under microgravity conditions in space supports the involvement of cortical microtubules in growth stimulation in space [27].

### 5.2. Morphogenesis

**Figure 5:**
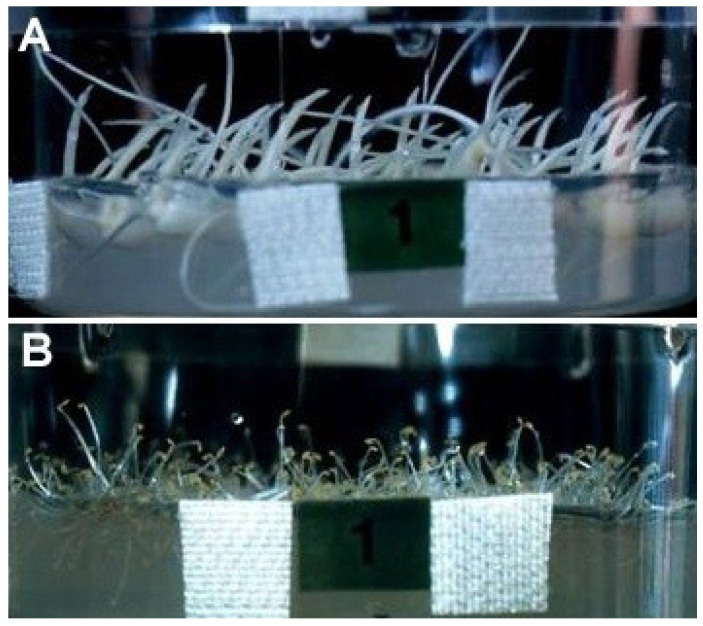
Rice seedlings (**A**) and Arabidopsis seedlings; (**B**) grown under microgravity conditions in space during the Space Shuttle STS-95 flight for 3 days. (**A**) short thick organs denote the shoots, and long thin ones denote the roots. All rice shoots bent toward the seed (to the right), and some roots emerged into the air; (**B**) the roots grew along the surface of the medium, and only the shoots are visible. Some shoots grew into the medium.

The changes in the morphology of plant seedlings have also been analyzed in space. The results obtained to date generally indicate that both shoot organs and roots of plant seedlings show automorphogenesis in space, as on the clinostat. The automorphogenesis of shoot organs in space was first reported in wheat coleoptiles [28]. In space, wheat coleoptiles show automorphic curvature, described as nastic curvature, away from the seed (caryopsis), and this bending angle decreases as the coleoptile age. Pea epicotyls also show automorphic curvature in space [29], as on the 3-D clinostat [3]. A detailed analysis of the automorphogenesis of space-grown rice and Arabidopsis shoots was conducted in the RICE experiment during the Space Shuttle STS-95 flight [30]. In space, rice coleoptiles bent toward the seed (Figure 5A). This bending consists of an inclination in the basal region and curvature in the elongating region. Both of these angles in rice coleoptiles grown in space were similar to those of rice grown on the 3-D clinostat [30]. On the other hand, Arabidopsis hypocotyls in space elongated in various directions, with approximately 10% even growing into the medium, without clear automorphic curvature (Figure 5B). Thus, the automorphogenesis of shoot organs in space consists of altered growth direction and automorphic curvature, as on the clinostat. In a number of space experiments, seedlings did not always show the typical automorphogenesis [22,29,31,32], which may have been caused by the reasons discussed above. Thus, the careful control of experimental conditions is required to observe and analyze automorphogenesis in space.

The morphogenesis of plant roots under microgravity conditions in space is much more diverse than that of shoots. The roots of garden cress cultivated in space during the D1 spacelab experiment grew in a direction forming a constant angle with the seed axis, with some degree of circumnutation [33]. However, garden cress showed random root movement in another experiment [34]. The difference in these results may be due to the age of roots. Lentil roots grown in space show a constant angle with respect to the baseline after strong curvature and straightening [35,36], as has been observed in garden cress roots. In space, Arabidopsis roots show waving, skewing to one direction, and coiling [37,38,39]. In the RICE experiment, we also analyzed the automorphogenesis of rice roots [30]. Rice roots elongated in various directions in space; more than 20% of these roots emerged into the air. However, the directions were not completely random, and two-thirds of the roots formed a constant mean angle of approximately 55° with the perpendicular baseline (Figure 5A). Similar results were obtained for rice plants grown on the 3-D clinostat.

The mechanism of differential growth during automorphic curvature was also examined with rice coleoptiles grown in space [40]. The dorsal, convex sides of rice grown in space showed higher mechanical extensibility of the cell wall than did the concave sides, as on the clinostat. The difference in the cell wall extensibility was highly correlated with the angle of curvature of rice coleoptiles. Additionally, the convex sides contained lower levels of cell wall polysaccharides with lower molecular masses, as well as higher breakdown activities, than did the concave sides. These results suggest that the uneven modifications of cell wall polysaccharide metabolism cause the automorphic curvature in rice coleoptiles under microgravity conditions.

## 6. Conclusions

Elongation growth of the shoot organs and roots is stimulated under true microgravity conditions in space. Similar modification of growth anisotropy is induced in some aquatic plants grown under submergence. Elongation growth is suppressed with increasing gravitational acceleration and varies in proportion to the logarithm of the magnitude of gravitational acceleration in the range from microgravity to hypergravity (Figure 4). On the other hand, plant organs show automorphogenesis in space, which may be masked by gravimorphogenesis on earth, except when growing on a clinostat. Thus, plants undergo significantly different growth and morphogenesis under space conditions. Plant growth may not always be good, under conditions in which automorphogenesis occurs, but this difficulty could be compensated by partial or fractional gravity. In this context, the gravity conditions on the Moon (0.17 *g*) and Mars (0.38 *g*) may be advantageous to plant life, as recently discussed by Kiss [41].

The cell wall plays a central role in the regulation of growth and morphogenesis in plants and is involved in the modifications of these events in space. The stimulation of elongation growth in space is caused by cell wall loosening, which is brought about by diverse changes in the metabolic turnover of cell wall constituents. Also, the automorphic curvature of stem organs in space may be caused by the inherent difference in the cell wall extensibility between the convex and concave sides, which is induced by the uneven modification of the metabolism of cell wall constituents. In space, the expression of the genes encoding the proteins responsible for cell wall metabolism, cytoskeleton formation, and plant hormone metabolism is greatly modified [27,42], which may induce changes in cell wall properties. On land, plants must develop a tough cell wall to resist gravitational acceleration, often employing more than 90% of their dry matter and energy consumption for this purpose. Under microgravity conditions in space, plants are set free from the strong constraint of gravity.

The ancestors of plants were born in the sea and evolved in earth environment. After these ancestors first emerged on land, approximately 450 million years ago in the mid-Ordovician, they developed sophisticated mechanism of gravity resistance [43,44]. In space, plants will experience entirely new conditions. The study of biology to date may be considered “1 *g* biology”, because it concerns only earth organisms under earth conditions. By assessing the universality of biological findings on the mechanism of earth life with spacefaring organisms in a space environment, biology itself may become further universal. In addition, space biology may contribute to efficient plant production, which is indispensable for human life not only in space but also on earth.
